# Transcriptional Repressive H3K9 and H3K27 Methylations Contribute to DNMT1-Mediated DNA Methylation Recovery

**DOI:** 10.1371/journal.pone.0016702

**Published:** 2011-02-08

**Authors:** Chun-Ming Wong, Carmen Chak-Lui Wong, Yeung-Lam Ng, Sandy Leung-Kuen Au, Frankie Chi-Fat Ko, Irene Oi-Lin Ng

**Affiliations:** State Key Laboratory for Liver Research and Department of Pathology, Li Ka Shing Faculty of Medicine, University of Hong Kong, Hong Kong, Special Administrative Region, People's Republic of China; Texas A&M University, United States of America

## Abstract

DNA methylation and histone modifications are two major epigenetic events regulating gene expression and chromatin structure, and their alterations are linked to human carcinogenesis. DNA methylation plays an important role in tumor suppressor gene inactivation, and can be revised by DNA methylation inhibitors. The reversible nature of DNA methylation forms the basis of epigenetic cancer therapy. However, it has been reported that DNA re-methylation and gene re-silencing could occur after removal of demethylation treatment and this may significantly hamper the therapeutic value of DNA methylation inhibitors. In this study we have provided detailed evidence demonstrating that mammalian cells possess a bona fide DNA methylation recovery system. We have also shown that DNA methylation recovery was mediated by the major human DNA methyltransferase, DNMT1. In addition, we found that H3K9-tri-methylation and H3K27-tri-methylation were closely associated with this DNA methylation recovery. These persistent transcriptional repressive histone modifications may have a crucial role in regulating DNMT1-mediated DNA methylation recovery. Our findings may have important implications towards a better understanding of epigenetic regulation and future development of epigenetic therapeutic intervention.

## Introduction

DNA methylation plays an important role in epigenetic transcriptional control. In mammalian genome, DNA methylation is established and maintained by the activity of DNA methyltransferases (DNMTs). DNMT3A and DNMT3B are known as *de novo* methyltransferases and are able to transfer methyl groups to unmethylated CpG dinucleotides [1], [2]. Activation of DNMT3A and DNMT3B during embryonic development establishes the DNA methylation pattern, which is essential for cell fate determination, as well as gene imprinting and X-chromosome inactivation [3], [4]. On the other hand, in somatic cells, maintenance of DNA methylation during DNA replication is carried out in a semi-conservative manner by the activity of DNMT1, which shows a higher affinity to hemimethylated DNA template and is physically associated with PCNA in the replication fork [1], [5]. This model provides a simple and elegant explanation for the inheritability of DNA methylation information. Recently, growing evidence has, however, indicated that the DNA methylation machinery is in fact more complicated. For example, it has been demonstrated that DNMTs physically bind to several histone modifiers including histone deacetylases (HDACs) [6], [7], SUV39H1 [8] and EZH2 [9]. The formation of multi-component epigenetic regulatory complex suggests that DNA methylation and histone modification machineries function in a highly cooperative manner in regulating chromatin structure and gene expression.

Epigenetic gene silencing, particularly DNA hypermethylation, has been recognized as an alternative alteration besides mutations and deletions in the “two hits” inactivation of tumor suppressor genes. Epigenetic gene silencing is a reversible process. Numerous studies have demonstrated that treatment of DNA methylation inhibitors such as 5-Aza-deoxycytidine (5-Aza-dC) can robustly reactivate the expression of epigenetically silenced tumor suppressor genes [4]. These findings form the basis of the therapeutic use of DNA methylation inhibitors, leading to the recent development of epigenetic therapy in cancer treatment [10]. Theoretically, pharmacologically demethylated CpG dinucleotides are inheritable and will be preserved upon DNA replication, unless secondary *de novo* DNA methylation takes place [11], [12]. Although DNA re-methylation and gene re-silencing after 5-Aza-dC treatment has been reported [12], [13], a fundamental question remains unanswered. This is because the DNA re-methylation process reported could simply be due to a selection artifact caused by the growth advantage of cells that were resistant to 5-Aza-dC treatment or might have occasionally escaped from DNA demethylation. Therefore, it is of crucial significance to investigate the detailed mechanisms of DNA re-methylation. Herein, we have provided strong evidence to demonstrate that DNA methylation recovery is a bona fide biological mechanism in mammalian cells and have revealed the indispensable role of DNMT1 in this DNA re-methylation. Our results also indicate that DNA methylation recovery was closely associated with transcriptional repressive H3K9 and H3K27 tri-methylations. These findings may have important implications to a better understanding of epigenetic regulation and future development of epigenetic therapeutic intervention.

## Materials and Methods

### Cell lines and 5-Aza-dC treatment

Cancer cell lines used in this study were obtained from Shanghai Institute of Cell Biology (SMMC-7721) or ATCC (HeLa). DNMT knock-out cell lines (1KO, 3bKO and DKO) and their parental HCT116 cells were kindly provided by Prof. B. Vogelstein, Johns Hopkins University School of Medicine, Baltimore, MD [14], [15]. SMMC-7721 and Hela cells were maintained in DMEM-high glucose (Gibcol), supplemented with 10% FBS (Invitrogen). Parental and DNMT KO HCT116 cell lines were grown in McCoy 5A medium (Sigma) and supplemented with 10% FBS. 5-Aza-dC (Sigma) was dissolved in 50% acetate and stored at -80°C until use. For 5-Aza-dC treatment, 3×10^4^ cells were seeded onto 60-mm dishes and treated with 5-Aza-dC at either 5 µM (HCT116 and HeLa) or 10 µM (SMMC-7721) for 4 days. 5-Aza-dC was replenished daily during the treatment. At Day 4, 5-Aza-dC was removed from the culture, and 5-Aza-dC treated cells were washed with PBS and allowed to recover in normal culture medium.

### RNA extraction and RT-PCR

Total RNA was extracted by TRIZOL reagent, according to the manufacturer's instructions (Life Technologies). cDNA was synthesized from 1 µg of total RNA by GeneAmp RNA PCR Kit with random hexamer primers in a 20 µL reaction mixture (Applied Biosystems). Semi-quantitative RT-PCR was carried out in 20 µL reaction containing 1X PCR buffer, 1X CG RICH buffer (Roche), 0.8 mM dNTP, 2 mM MgCl_2_, 0.5 µM of each primers, 0.1U AmpliTaq Gold and 2 µL of cDNA (Applied Biosystems). The PCR was terminated at the exponential phases: 30 cycles for *DLC-1* and 18 cycles for *GAPDH* including one cycle of hot-start at 95°C for 12 min, followed by amplification at 94°C for 30 s, 55°C for 30 s, 72°C for 45 s, and a final extension at 72°C for 10 min. Quantitative RT-PCR was carried out in 20 µL reaction mixture containing 1X TaqMan Master mix, 1X gene-specific TaqMan porbe and 1 µL cDNA. The PCR reaction was performed with ABI 7900HT system (Applied Biosystems) at the following conditions: 50°C for 2 min, 95°C for 10 min followed by 40 cycles of 95°C for 15 s, 60°C for 1 min. Gene expression was normalized against endogenous control *HPRT* by ΔCt _[Target – HPRT]_. Relative gene expression was determined by ΔΔCt _[Control – Test]_ and expressed as fold change relative to the corresponding control sample (i.e. 2^-.ΔΔCt^). PCR primers and TaqMan Probes used in this study are listed in Table S1 and Figure S1.

### Immunoblotting

Protein was extracted by NET-NP40 buffer in the presence of Complete™ Protease Inhibitor Cocktail (Roche). 20 µg of protein was separated in 8% SDS-PAGE and transferred to PVDF membrane (Amersham) for immunoblotting. The membrane was probed by anti-DLC1 antibody (1∶200, BD Biosciences) [16] and β-actin (Sigma) followed by incubation with anti-mouse IgG (GE Healthcare). Protein expression was detected with the ECL™ detection system (GE Healthcare) according to the manufacturer's protocol.

### DNA extraction and methylation analysis

High-quality genomic DNA was extracted by phenol/chloroform after proteinase K treatment. Sodium bisulfite treatment was carried out using the CpGenome DNA modification kit (Chemicon). Two µL of bisulfite treated DNA was amplified by PCR with primers specific for methylated and unmethylated alleles of the *DLC-1* gene. Reaction were carried out at the following conditions: hot start at 95°C for 12 min, followed by 32 cycles of 94°C for 30 s, 58°C for 30 s and 72°C for 30 s, and final extension at 72°C for 10 min. Semi-quantitative analysis was performed by determining the band intensity using AlphaEaseFC software (Alpha Innotech). For Pyrosequencing, 5 µL disfulite treated DNA first amplified in a 50 µL reaction with CG RICH solution (Roche) at the following PCR cycles: 95°C for 12 min, followed by 45 cycles of 94°C for 30 s, 55°C for 30 s and 72°C for 30 s, and final extension at 72°C for 10 min. Biotinated single strand PCR product was purified with Streptavidin coated Sepharose beads and subjected to Pyrosequencing in PyroMark ID system as manufacture's instruction (Biotage). Primer sequences are listed in Table S1 and Figure S1.

### Establishment of 5-Aza-dC-recovered clones

SMMC-7721 cells were treated with 5-Aza-dC as described above. At Day 4, 5-Aza-dC treated cells were trypsinized, diluted, plated onto 100-mm culture dishes and recovered in normal culture medium for two weeks. Colonies formed by single 5-Aza-dC-recovered clones were isolated from the culture dishes using a Cloning cylinder (Bellco Biotechnology). The expression and methylation level of hypermethylated genes were analyzed as mentioned above.

### Chromatin immunoprecipitation (ChIP) assay

2×10^7^ cells of each parental SMMC-7721 and its various derived clones were used for ChIP assay. Chromatin was crosslinked with formaldehyde and sonicated to an average size of 200–1000 bp. ChIP assay was performed with EZ chromatin immunoprecipitation kit according to the manufacturer's protocol (Upstate biotechnology, Lake Placid, NY). 1 µg of rabbit antibody against tri-methylated H3K4, tri-methylated H3K9 and tri-methylated H3K27, respectively, were mixed with sheared chromatin and incubated at 4°C overnight. Chromatin-antibody complexes were then precipitated with Salmon Sperm DNA/Protein A agarose. Reagents and antibodies used in ChIP assay were obtained from Upstate. Real-time PCR amplification was carried out with Power SYBR Green PCR Master Mix according to manufacturer's instructions (Applied Biosystems, Foster City, CA). PCR cycles are identical to the quantitative RT-PCR used for gene expression analysis described above. Primer sequences are listed in Table S1 and Figure S1. Data are presented as “Relative enrichment” by the equation 2^[ΔCt (No Antibody –Input) - ΔCt (Target – Input)]^.

## Results

### Restoration of DNA methylation after removal of demethylating agent was a general phenomenon

**Figure 1:**
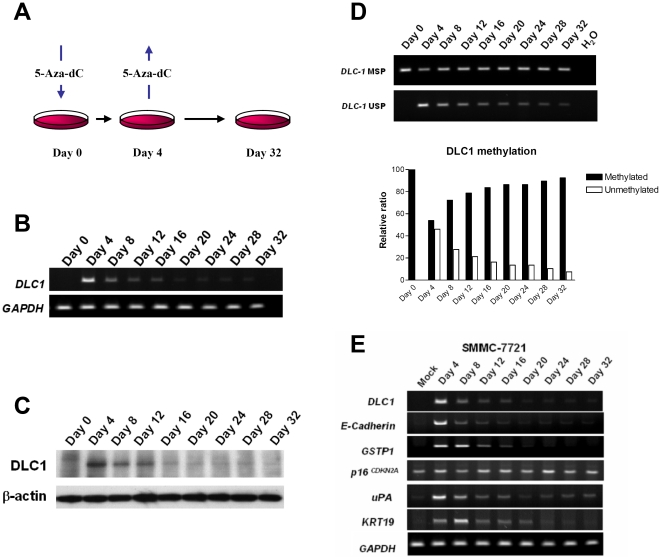
DNA re-methylation after 5-Aza-dC treatment. (A) Schematic outline of the experimental design. SMMC-7721 cells were treated with 5-Aza-dC at 10 µM for 4 days to induce global demethylation. At Day 4, 5-Aza-dC was removed and cells were replenished with normal culture medium (DMEM-high glucose, supplemented with 10% FBS). 5-Aza-dC treated cells were allowed to recover in the absence of 5-Aza-dC for an additional 4 weeks. *DLC1* mRNA (B) and protein (C) expression in SMMC-7721 was gradually re-silenced when released from 5-Aza-dC treatment at Day 4. (D) Consistent with re-silencing of *DLC1* expression, SMMC-7221 cells gradually re-acquired *DLC1* promoter methylation after removal of 5-Aza-dC treatment, as revealed by methylation-specific PCR. MSP: methylation-specific PCR, USP, unmethylation-specific PCR. Semi-quantitative analysis was done by AlphaEaseFC software (Alpha Innotech, San Leandro, CA). (E) In addition to *DLC1*, gene re-silencing was also observed in multiple hypermethylated genes, including *E-cadherin*, *GSTP1*, *uPA* and *KRT19*. In contrast, the expression level of unmethylated tumor suppressor gene *p16^CDKN2A^* was not affected and remained constant throughout.

**Figure 2:**
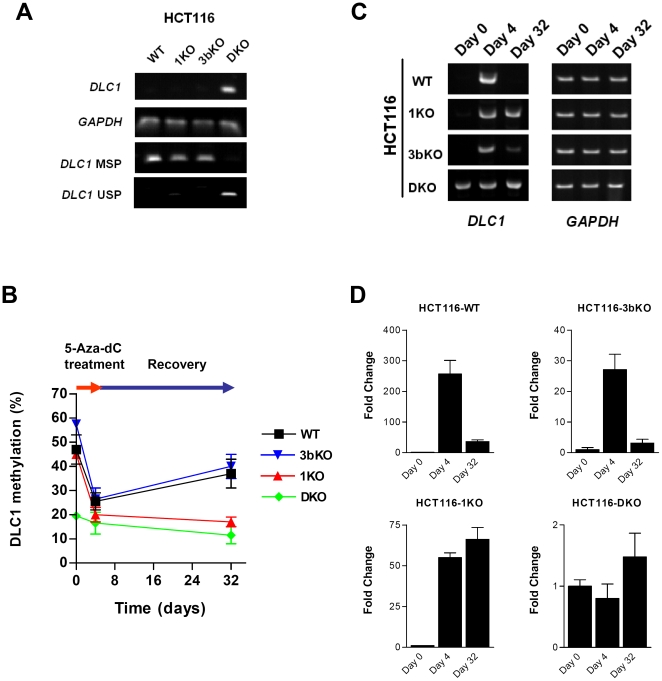
DNMT1 was indispensable for DNA methylation recovery. (A) *DLC1* was silenced by DNA hypermethylation in parental HCT116 cells but was substantially expressed in double knockout (DKO) cells. (B) Re-methylation of the *DLC1* CpG island in HCT116 cells after 5-Aza-dC treatment was quantitatively determined by pyrosequencing. DNMT1 knockout (1KO) cells failed to restore *DLC1* promoter methylation. In contrast, DNMT3B knockout cells (3bKO) re-acquired *DLC1* promoter methylation as efficiently as parental HCT116 cells. Consistently, 1KO and DKO cells were unable to re-silence *DLC1* gene expression, in contrast to that observed in parental HCT116 and 3bKO cells. Re-silencing of *DLC1* mRNA expression in HCT116 cells was determined by semi-quantitative (C) and real-time quantitative RT-PCR (D). *DLC1* expression was normalized against *GAPDH* and *HPRT,* respectively. Data are represented as mean ± SEM from three independent experiments.

In this study, first, we made use of the DNA demethylating action of 5-Aza-dC to induce global DNA demethylation in a hepatocellular carcinoma (HCC) cell line, SMMC-7721, and investigated if the cells could restore their DNA methylation information when the drug was removed (Fig. 1A). We found that the cancer cells were able to restore their DNA methylation information disturbed by 5-Aza-dC treatment. *DLC1* (Deleted in liver cancer 1), a hypermethylated tumor suppressor gene in primary HCCs and in SMMC-7721 (Figure S2) [17], was drastically re-expressed upon 5-Aza-dC treatment. However, this re-expression rapidly disappeared once 5-Aza-dC was removed at Day 4 (Fig. 1B and 1C). Concomitant with gene re-silencing, *DLC1* promoter gradually re-acquired its DNA methylation (Fig. 1D). Similar gene re-silencing phenomenon was also observed in other known hypermethylated tumor suppressor genes (*E-Cadherin* and *GSTP1*), oncogenes (*uPA*) and tissue-specific genes (*KRT19*) (Fig. 1E). In contrast and consistent with its unmethylation status, the expression level of tumor suppressor gene *p16^CDKN2A^* was not affected throughout the study (Fig. 1E). Apart from SMMC-7721 HCC cell line, DNA re-methylation was also consistently observed in other cancer cell lines, including HCT116 (Fig. 2) and HeLa (Figure S3), indicating that it was not a cell type-specific phenomenon. In agreement with our observation of DNA re-methylation in hypermethylated promoters, similar findings have previously been reported in *Alu* repeats [12], [13]. Thus, data from previous studies and the present one, when taken together, strongly suggest that DNA re-methylation following demethylating treatment is a general phenomenon. Although it has not been investigated in detail, this phenomenon has potential significance in understanding the epigenetic regulation system and therapeutic use of DNA methylation inhibitor in treating human cancers and thus prompted us to further determine its underlying mechanisms.

### DNMT1, instead of DNMT3B, was indispensable for DNA methylation recovery

DNA methylation on CpG dinucleotide is mainly regulated by the activity of DNMTs. Previous work has demonstrated that *de novo* methyltransferase DNMT3B is essential to the re-methylation of repetitive sequences that are demethylated during the early stages of embryogenesis [18]. We queried whether the aforementioned DNA re-methylation was a result of *de novo* DNA methylation and mediated by the activity of DNMT3B and whether other DNMTs were involved. To address this query, we extended our study with a series of HCT116 cells lines in which *DNMT1*, *DNMT3B*, or both *DNMT1* and *DNMT3B* were deleted through homologous recombination [14], [15]. We observed that the tumor suppressor gene *DLC1* was silenced by DNA hypermethylation in parental HCT116 cells but was substantially expressed in double knockout (DKO) cells (Fig. 2A) or re-expressed upon 5-Aza-dC treatment (Fig. 2C and 2D). Surprisingly, we found that DNMT3B knockout cells (3bKO), when released from 5-Aza-dC treatment, re-acquired *DLC1* promoter methylation as efficiently as parental HCT116 cells (Fig. 2B). In contrast, DNMT1 knockout (1KO) and double knockout (DKO) cells failed to restore *DLC1* promoter methylation (Fig. 2B) and consistently, were unable to re-silence the *DLC1* gene expression (Fig. 2C and 2D). Therefore, our findings clearly indicate that, different from *de novo* methylation during the early stages of embryogenesis, DNA methylation recovery in somatic cells following 5-Aza-dC treatment was mediated by DNMT1, and not by the conventional *de novo* DNA methyltransferase, DNMT3B.

### 5-Aza-dC-recovered cells remained sensitive to 5-Aza-dC-induced DNA demethylation

**Figure 3:**
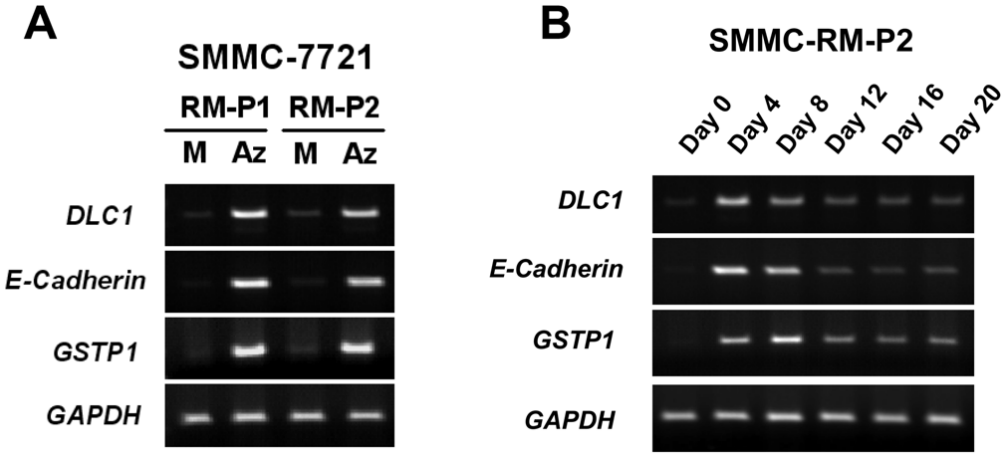
5-Aza-dC-recovered cells were sensitive to 5-Aza-dC induced derepression. To exclude the potential artifact of DNA re-methylation caused by 5-Aza-dC-resistant cells, two 5-Aza-dC-recovered sub-lines (SMMC-7721 RM-P1 and P2) were established from pooled 5-Aza-dC-recovered population of SMMC-7721. (A) The expression of re-methylated tumor suppressor genes, *DLC1*, *E-Cadherin* and *GSTP1*, could successfully be activated once again in these recovered cells by re-administration of 5-Aza-dC. M, Mock; Az, 5-Aza-dC. (B) 5-Aza-dC-recovered cells retained the ability to re-silence *DLC1* after being released from 5-Aza-dC treatment. These data indicate that 5-Aza-dC-recovered cells remained sensitive to 5-aza-dC-induced derepression and re-silencing Thus, ruled out the possibility of growth selection artifact caused by the expansion of 5-Aza-dC-resistant sub-populations.

One major concern regarding the methylation recovery observed in our system was whether the aforementioned DNA re-methylation was merely reflecting a selection attributed to the growth advantage of 5-Aza-dC-resistant cells. To address this query, we established two independent SMMC-7721 sub-lines from the pooled 5-Aza-dC-recovered populations (SMMC-7721 RM-P1 and P2). We found that these 5-Aza-dC-recovered sub-lines, upon 5-Aza-dC treatment as well as during 5-Aza-dC recovery, responded similarly as their parental SMMC-7721 cells. Upon re-administration of 5-Aza-dC, hypermethylated genes, including *DLC1*, *E-cadherin* and *GSTP1*, were successfully re-expressed in these 5-Aza-dC-recovered cells (Fig 3A). In addition, these genes were gradually re-silenced upon relief of 5-Aza-dC treatment (Fig. 3B). Our findings therefore indicate that these recovered cells remained sensitive to 5-Aza-dC-induced demethylation and retained the ability of DNA methylation recovery. Thus, we concluded that the DNA re-methylation after 5-Aza-dC treatment was not an artifact caused by the expansion of 5-Aza-dC-resistant sub-populations.

### Incomplete DNA re-methylation after 5-Aza-dC treatment

**Figure 4:**
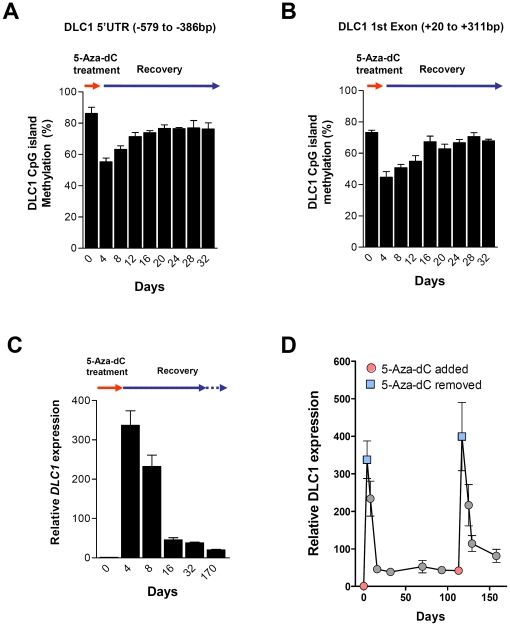
Incomplete DNA methylation recovery after 5-Aza-dC treatment. *DLC1* CpG island methylation and mRNA expression in SMMC-7721 cells during the 5-Aza-dC treatment (Day 0 to Day 4) and recovery (Day 4 to Day 170) were determined. Approximately 10% of the overall DNA methylation on the *DLC1* 5′UTR (A) and 1^st^ exon (B) was permanently lost after 5-Aza-dC treatment. Quantitatively analysis was done by pyrosequencing, and the average methylation of 10 and 8 CpG dinucleotides on 5′UTR and first exon, respectively, was obtained from three independent experiments and is presented as mean ± SEM. (C). Consistently, a low level of residual *DLC1* gene expression in 5-Aza-dC-recovered cells could be detected in SMMC-7721 cells (up to 170 days). (D). Residual *DLC1* gene expression was significantly higher (P = 0.0010, t-test) in the 5-Aza-dC-recovered cells that were re-administered with 5-Aza-dC (comparing day 16 and day 129, i.e. 12 days after 1^st^ and 2^nd^ 5-Aza-dC treatment), suggesting that multiple administrations may be required to ensure complete demethylation of tumor suppressor genes following treatment with 5-Aza-dC. Opened circles and rectangles indicate the start and the end of 5-Aza-dC treatment, respectively (Mean ± SEM, N = 4).

We also observed that, although cancer cells were capable of re-silencing 5-Aza-dC-demethylated genes, low levels of residual expression of these normally silenced genes were detected in the 5-Aza-dC-recovered cells (Fig. 1E). We therefore quantitatively monitored the DNA methylation level of *DLC1* gene throughout the 5-Aza-dC treatment and recovery process. In the SMMC-7721 cells, using pyrosequencing to quantify the extent of methylation, approximately 10% of the overall DNA methylation on the *DLC1* CpG islands (on 5′UTR and 1^st^ exon regions) was permanently lost after 5-Aza-dC treatment (Fig. 4A and 4B). In fact, we could consistently detect a low level of *DLC1* gene expression in the 5-Aza-dC-recovered cells up to 170 days (Fig. 4C). This observation implies that the repressive epigenetic information disturbed by 5-Aza-dC treatment could not be completely restored. Interestingly, although 5-Aza-dC-recovered cells retained the ability of DNA methylation recovery upon repetitive demethylation, the residual gene expression level was significantly higher in the recovered cells that had undergone repetitive 5-Aza-dC treatment (Fig. 4D). In light of above findings, we speculated that multiple administrations may be required to ensure complete re-activation of tumor suppressor genes following treatment with 5-Aza-dC and perhaps other DNA methylation inhibitors. This may have implications on the administration and efficacy of DNA methylation inhibitors in cancer treatment.

### Consistent enrichment of H3K4-tri-methylation among heterogeneous re-methylated *DLC1* locus ruled out the possibility of stochastic DNA demethylation escape

**Figure 5:**
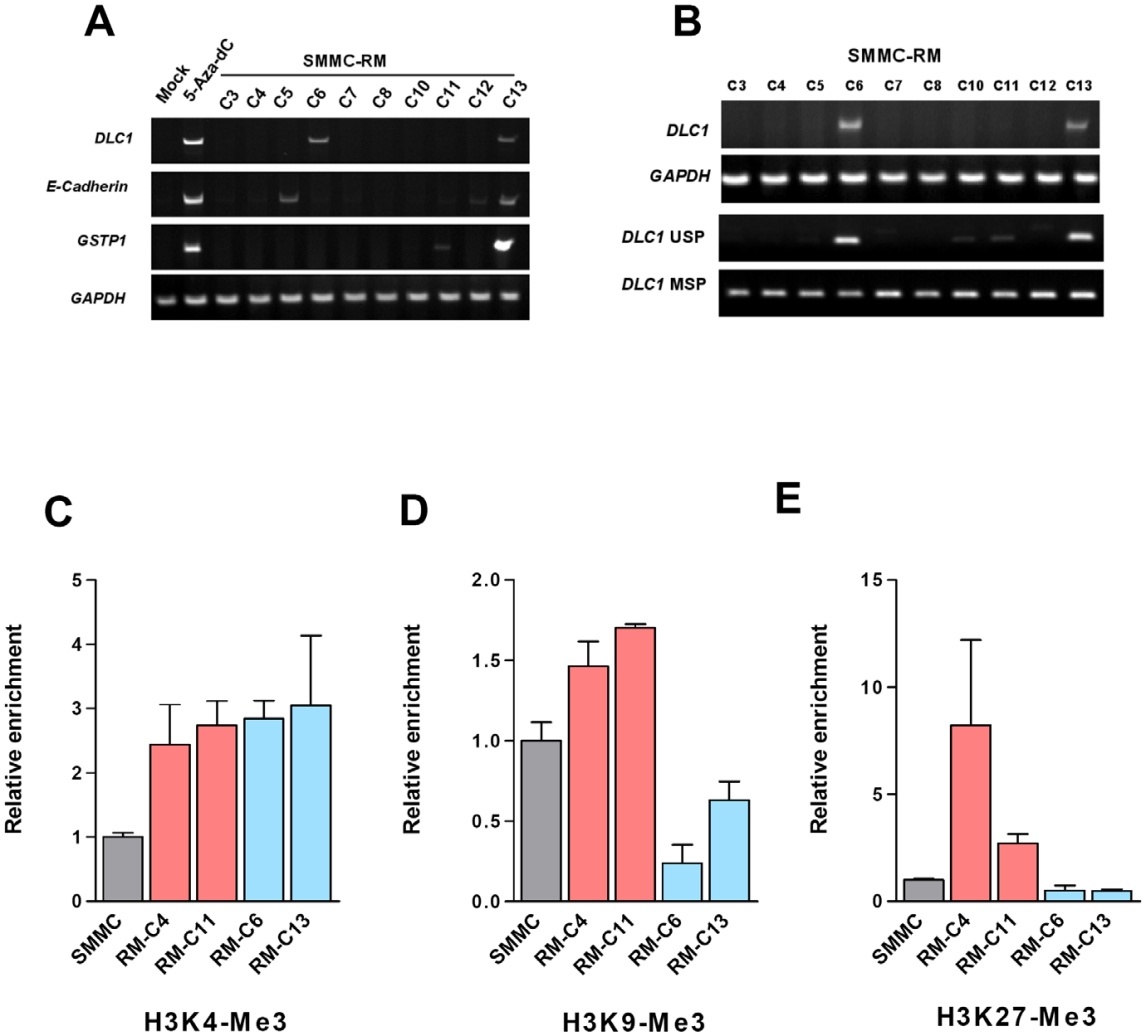
Differential histone modifications associated with heterogeneous DNA methylation recovery. Individual 5-Aza-dC-recovered clones (SMMC-RM-C3 to C13) were established by clonal expansion of SMMC-7721 cells after 5-Aza-dC treatment. (A) mRNA expression of normally hypermethylated tumor suppressor genes (*DLC1*, *E-Cadherin* and *GSTP1*) in each of the 5-Aza-dC-recovered clones was determined by semi-quantitative RT-PCR. (B) Methylation of *DLC1* gene in individual 5-Aza-dC-recovered clones was determined by methylation-specific PCR. Incomplete gene re-silencing of *DLC1*, *E-Cadherin*, and *GSTP1* was found in multiple 5-Aza-dC-recovered clones, but the genes involved varied among individual clones. (C) H3K4 tri-methylation (H3K4-me3) was considerably enriched on *DLC1* promoter in all 5-Aza-dC-recovered clones when compared with their parental SMMC-7721 cells, indicating that the *DLC1* locus in all 5-Aza-dC-recovered clones tested had undergone DNA demethylation during 5-Aza-dC treatment. (D) H3K9 tri-methylation (H3K9-me3) was preserved in *DLC1* re-methylated clones, whereas it was significantly reduced in the *DLC1* hypomethylated clones (i.e. RM-C6 and C13). (E) H3K27-me3 level was significantly enriched on the *DLC1* promoter exclusively in those re-methylated clones (RM-C4 and C11), whereas the H3K27-me3 level remained unchanged in those hypomethylated clones (RM-C6 and C13). These findings suggest that preserved H3K9-me and enrichment of H3K27-me3 level were associated with DNA methylation recovery mediated by DNMT1. Histone modifications on *DLC1* promoter region were revealed by ChIP assay using antibody against H3K4-me3, H3K9-me3 and H3K27-me3, respectively. Quantitative data were obtained with real-time PCR and are presented as “Relative enrichment” (mean ± SEM). Data were obtained from three independent repeats.

By investigating a series of clonally expanded 5-Aza-dC-recovered cells, we found substantial gene expression as a result of incomplete DNA methylation recovery in multiple 5-Aza-dC-recovered clones. The genes showed incomplete DNA re-silencing including *DLC1*, *E-Cadherin*, and *GSTP1*, but exhibited a differential pattern among individual clones (i.e. RM-C6 and C13 for *DLC1*; RM-C5, C12 and C13 for *E-Cadherin* and RM-C11 and C13 for *GSTP1*, respectively) (Fig. 5A and 5B). Hence, the incomplete DNA methylation recovery was apparently heterogeneous with regard to individual genes involved. This observation provided an explanation accounting for the low residual gene expression seen in 5-Aza-dC-recovered cells. However it also raised the possibility that DNA methylation recovery might be an artifact of selection advantage for cells that occasionally escaped from DNA demethylation during 5-Aza-dC treatment. To exclude this possibility, we compared the H3K4 tri-methylation level on the *DLC1* locus among the 5-Aza-dC-recovered clones with reference to their DNA methylation status. It has been reported that H3K4 methylation could be enriched upon 5-Aza-dC treatment, likely a secondary event following the promoter demethylation and gene re-expression [19], [20], [21]. Consistent with this notion, in SMMC-7721 cells we also observed a remarkable enrichment of H3K4 methylation at DLC1 locus upon 5-Aza-dC treatment (Figure S4). Therefore, H3K4 methylation level could be used as a trace mark for locus that had undergone DNA demethylation. Two *DLC1*-remethylated clones (RM-C4 and C11) and two *DLC1*-hypomethylated clones (RM-C6 and C13) were selected for investigation. Using chromatin immunoprecipitation (ChIP) assay, we found that regardless of their status of DNA methylation recovery and gene expression level, H3K4 tri-methylation (H3K4-me3) was considerably enriched on *DLC1* promoter in all 5-Aza-dC-recovered clones when compared with their parental SMMC-7721 cells (Fig. 5C). This finding indicate that the *DLC1* locus in all 5-Aza-dC-recovered cells tested had undergone DNA demethylation during 5-Aza-dC treatment and thus ruled out the possibility of growth selection of stochastic demethylation escaped cells.

### H3K9 and H3K27 tri-methylation were associated with DNA methylation recovery

The heterogeneous pattern of DNA methylation recovery strongly suggested that the repressive epigenetic information disturbed by 5-Aza-dC treatment might not be completely restored. Having excluded the possibility of growth selection of cells which might have stochastically escaped demethylation, we therefore considered another possible explanation that differential DNA methylation recovery could be determined by local histone modification marks. We hypothesized that stable transcriptional repressive histone modifications might be preserved in those re-methylated locus and contribute to the DNA methylation recovery. Herein, we found that in contrast to H3K4 methylation, H3K9 tri-methylation (H3K9-me3) was preserved in *DLC1* re-methylated clones, whereas it was significantly reduced in the *DLC1* hypomethylated clones (i.e. RM-C6 and C13) (Fig. 5D). The concurrence of the loss of H3K9-me3 and the absence of DNA re-methylation in these expanded clones implicate a crucial role of H3K9-me3 in DNA methylation recovery. Next, we queried whether another stable repressive histone modification, H3K27 tri-methylation (H3K27-me3), might also play a role in DNA methylation recovery. In this regard, we observed a notable increase in H3K27-me3 level on the *DLC1* promoter exclusively in those re-methylated clones (RM-C4 and C11), whereas the H3K27-me3 level remained unchanged in those hypomethylated clones (RM-C6 and C13) as compared with their parental SMMC-7721 cells (Fig. 5E). Our findings therefore implied that increased H3K27-me3 level was associated with DNA methylation recovery mediated by DNMT1.

## Discussion

The contribution of epigenetic changes in tumor suppressor gene inactivation and human carcinogenesis has received much attention in the past years [4], [22], [23]. Among all epigenetic alterations, DNA hypermethylation on the promoter region of tumor suppressor genes probably is most well characterized. Unlike genetic alterations such as gene mutation and chromosomal amplification/deletion, DNA hypermethylation is considered as a reversible process and this has therefore rendered the basis of cancer epigenetic therapy [10], [24], [25], [26]. Mounting pre-clinical evidence has demonstrated that treatment of DNA methylation inhibitors can successfully restore the expression of hypermethylated tumor suppressor genes and inhibit cancer cell growth. However, recent studies have indicated that those demethylated genes or loci can undergo re-methylation once released from demethylating treatment [25], [26]. This observation has added new insight towards the better understanding of the complicated epigenetic regulation system and may have potential implication for clinical use of DNA methylation inhibitors in cancer treatment. However, the underlying mechanisms of this DNA methylation recovery process remain largely unknown and further investigation is much wanted.

In this study, we employed the commonly used DNA methylation inhibitor, 5-Aza-dC, to induce global DNA demethylation in human cancer cells. Consistent with previous studies, we observed that multiple DNA hypermethylated genes were robustly reactivated by 5-Aza-dC [27], [28]. However, these genes gradually underwent DNA re-methylation and were re-silenced once released from the treatment. Apparently, gene re-silencing is a genome-wide phenomenon. We showed that resilencing was not only found in well characterized tumor suppressor genes but also in those normally hypermethylated oncogenes and tissue specific genes. Based on our current knowledge about DNA methylation, it is reasonable to deduce that DNA methyltransferases may be involved in the DNA methylation recovery process. Although it has been hypothesized that *de novo* methyltransferase DNMT3A or DNMT3B might be the major player, this notion was not supported by our experimental data. With the widely used DNMT knock-out HCT116 cell models, we have shown that loss of DNMT1 alone was sufficient to abolish the DNA re-methylation. In contrast, loss of DNMT3B had no detectable effect on the DNA methylation recovery process. Similar finding has also been previously reported by Egger and colleagues [29].

It is important to note that global DNA methylation level was only slightly reduced in this particular DNMT1 KO cell line, indicating that its “maintenance” methyltransferase activity was basically preserved [14], [15]. The “maintenance” methyltransferase activity in this DNMT1 KO cell line could at least be partially explained by the recently identified truncated DNMT1 protein, which is an alternatively splicing product and could bypass the somatic deletion of *DNMT1* gene in this HCT116 cell line [29], [30]. This truncated DNMT1 protein retains its catalytic activity but lacks the PCNA-binding domain [31]. Recent work has shown that the PCNA-binding domain was essential in recruiting DNMT1 to DNA repair sites immediately after UV irradiation [32]. It is likely that the PCNA-binding domain may also important for this DNMT1-mediated DNA methylation recovery. Therefore lack of the PCNA-binding domain in this truncated DNMT1 may provide an explanation to account for the loss of DNA methylation recovery function in this DNMT1 KO cell line, despite the fact that the “maintenance” methylation was not significantly affected. Based on above findings, we propose that DNMT1 may possess two independent functions in maintaining DNA methylation integrity: on one hand, DNMT1 is responsible for maintaining DNA methylation pattern in the newly synthesized DNA strand after DNA replication; on the other, DNMT1 is also involved in DNA methylation recovery probably via its *de novo* methyltransferase activity [33].

In this study, we noticed that although the majority of 5-Aza-dC treated cells underwent DNA re-methylation upon drug relief, the DNA methylation recovery was however not a perfect process. Among the ten 5-Aza-dC recovery clones established from SMMC-7721 cells, two of them failed to re-acquire their DNA methylation level at *DLC1* promoter that was disturbed by 5-Aza-dC treatment. This incomplete DNA methylation recovery accounted for approximately 10% loss of overall DNA methylation level and consequently led to the presence of a low level of gene expression in 5-Aza-dC-recovered population. It is well documented that both unmethylated DNA and H3K4 hypermethylation are associated with transcriptionally active euchromatin, whereas densely methylated DNA and H3K9 hypermethylation are frequently found in transcriptionally repressive heterochromatin, suggesting that the two major layers of epigenetic regulation, DNA methylation and histone modification, are in fact functionally linked [22], [34], [35]. We therefore tested whether this DNA methylation recovery mediated by DNMT1 would be regulated by local repressive histone modifications. It has previously been shown that 5-Aza-dC treatment resulted in loss of H3K9-methylation and enrichment of both H3K9 acetylation and H3K4-methylation. These histone modifications were accompanied with DNA demethylation and re-expression of *hMLH1* gene in colon cancer cell lines [19]. Since currently there is no evidence indicating that 5-Aza-dC may have direct effect on histone modification, these histone modification changes likely occurred as a secondary event upon DNA demethylation and together facilitated the reactivation of epigenetic silenced genes. Indeed, we observed a consistent enrichment of H3K4 methylation at the normally DNA hypermethylated *DLC1* promoter in all 5-Aza-dC-recovered clones regardless of their DNA re-methylation status. This H3K4 methylation enrichment was consistent with those of the previous reports [19], [36] and indicated that *DLC1* promoter in all 5-Aza-dC-recovered clones had underwent DNA demethylation during 5-Aza-dC treatment.

Interestingly, previous study had indicated that the stable repressive marks (H3K9-me3 and H3K27-me3) remained unchanged upon 5-Aza-dC treatment. Inferred from this observation, it has been speculated that these stable repressive marks may be related to DNA methylation recovery [36]. However, two most recent studies reported that 5-Aza-dC treatment converted the transcriptional repressive histone modifications pattern to transcriptional active marks [20], [21]. To explore the role of repressive histone modifications on DNA methylation recovery and gene re-silencing, we compared the H3K9-me3 levels between 5-Aza-dC-recovered clones with or without DNA re-methylation. Our data showed that H3K9-me3 level was preserved in the majority of 5-Aza-dC-recovered clones that had exhibited DNA re-methylation at *DLC1* promoter and gene re-repression (as in RM-C4 and C11 clones). In contrast, we observed a significant decrease of H3K9-me3 level in the 5-Aza-dC-recovered clones that showed no DNA re-methylation. Our findings suggest that loss of H3K9-me3 caused by 5-Aza-dC treatment may impair the DNA re-methylation process. Consistent with this notion, mutation of K9 residue of histone 3 or H3K9 specific histone methyltransferase was found to induce gross DNA hypomethylation [37], [38]. Therefore, it is reasonable to believe that H3K9-me3 is essential for DNA methylation recovery. We reason that DNA demethylation caused by 5-Aza-dC treatment may eventually lead to re-establishment of local histone modifications and chromatin structure. Once this happens, the repressive epigenetic status may not be completely restored by the DNA methylation recovery system.

Interestingly, we also found a remarkable increase of H3K27-me3 at the *DLC1* promoter of 5-Aza-dC-recovered clones. H3K27-me3 enrichment was exclusively detected in those re-methylated clones whereas it remained unchanged in the two clones that showed no DNA re-methylation. This observation suggests that H3K27-me3 may also play a role in DNA methylation recovery. It is known that H3K27-me3 is catalyzed by a polycomb group protein EZH2 [39]. EZH2 is physically associated with human DNMT1, and depletion of EZH2 results in significant reduction of DNA methylation on the promoter region of *MYT1* gene [9]. More recently, significant enrichment of H3K27 methylation has been found in hypermethylated genes in human cancers and implicated in *de novo* DNA methylation [40], [41], [42]. All these lines of evidence suggest that the persistent H3K9-me3 and increased H3K27-me3 may serve as an initial signature for the recruitment of DNMT1 to demethylated loci and execute its DNA methylation recovery function.

The major concern of DNA re-methylation observed in previous studies is whether this phenomenon was merely reflecting an artifact ensued from growth selection rather than a meaningful biological mechanism. Herein, we have provided several lines of evidence to support the presence of DNA methylation recovery system in mammalian cells. First, DNA methylation recovery was consistently observed in the different cell lines that expressed functional DNMT1 but was completely abolished in the DNMT1 knock-out cell lines (HCT116 1KO and DKO). If DNA re-methylation were merely due to growth selection, this selection artifact should have applied to all cell lines tested regardless of their DNMT1 status. However, as discussed above, the function of DNMT1 was apparently involved in this DNA re-methylation process and thus provided mechanistic evidence to the DNA methylation recovery system. Second, 5-Aza-dC-recovered cells were as sensitive to 5-Aza-dC treatment as their parental cells, indicating that DNA re-methylation was unlikely caused by the expansion of 5-Aza-dC-resistant sub-populations. Third, the consistent enrichment of H3K4 me3 level at normally DNA hypermethylated loci provided clear evidence supporting the presence of DNA demethylation during the 5-Aza-dC treatment and excluded the involvement of stochastic DNA demethylation escape. The above lines of evidence, when taken together, strongly suggest that mammalian cells possess a bona fide DNA methylation recovery system and exclude the possibility of growth selection artifact. The biological significance of the aforesaid DNA methylation recovery system needs to be further investigated. Inferring from the observation that homologous deletion of DNMT1 significantly abolished the colony forming ability of HCT116 cells after 5-Aza-dC treatment (unpublished observation), we speculate that DNMT1-mediated DNA methylation recovery may have an important role in protecting the cells against unphysiological demethylation.

Our present findings on the DNA methylation recovery system also have potential implication in the clinical use of DNA methylation inhibitors in cancer treatment. Two major DNA methylation inhibitors, azacytidine (5-Aza-cytidine) and decitabine (5-Aza-deoxycytidine) have been approved by Food and Drug Administration (FDA) for treating myelodysplastic syndrome and have shown promising anti-cancer effects in various myeloid malignancies in recent clinical trials. However, the clinical outcome of these drugs in treating solid tumors was not totally satisfactory [26]. One possible reason is attributed to the instability of DNA methylation inhibitors in physiological conditions in that they became undetectable within a short time after administration [43], [44]. The 5-Aza-dC relief approach used in this system actually mimics the consequence of rapid elimination of DNA methylation inhibitors in clinical situation. In this scenario, cancer cells will take advantage of DNA methylation recovery system, resulting in re-silencing of DNA hypermethylated genes. In this regard, the efficacy of DNA methylation inhibitors in cancer treatment could be significantly improved if the DNA methylation recovery system could be suppressed or minimized. We have shown that, despite the finding that cancer cells possess DNA methylation recovery system, DNA methylation disturbed by 5-Aza-dC treatment could not be completely restored. More importantly, DNA demethylation accumulated upon repetitive treatment, likely attributable to the re-establishment of local histone modifications as discussed above. Thus our findings have provided experimental evidence to support the rationale of repetitive administration in cancer treatment in order to maximize the demethylating effect. Moreover, in light of the essential roles of H3K9-me3 and H3K27-me3 in DNMT1-mediated DNA methylation recovery demonstrated in this study, it can be inferred that combined treatment of DNA methylation inhibitors with SUV39H1 (H3K9-me3 specific histone methyltransferase) or EZH2 (H3K27-me3 specific histone methyltransferase) inhibitors may have potential advantage in suppressing this DNA methylation recovery and achieving enduring DNA demethylation. Small molecular inhibitors that specifically target SUV39H1 and EZH2 have been discovered recently [45], [46]. Pioneering studies have shown that both SUV39H1 and EZH2 inhibitors exhibited cancer suppressive effects *in vitro* [45], [46], [47]. We anticipate that their toxicity, anti-cancer activity and therapeutic potential will be extensively evaluated in the foreseeable future. It would be interesting to test whether such combined treatments may improve the clinical outcome of our current epigenetic therapy protocol. In summary, the findings presented here have provided mechanistic evidence towards the DNA methylation recovery system and may have potential implications for the development of new epigenetic therapeutic strategy.

## Supporting Information

Figure S1 **Position of primers used in expression and epigenetic studies.** Positions indicated above are reference to the putative *DLC1* transcription start site (TSS) according to the Genebank database NM_006094.
(PDF)

Figure S2 ***DLC1*** **methylation in normal liver cell line (MIHA) and liver cancer cell line (SMMC-7721).** DNA methylation at +45 to +336 position of *DLC1* gene was analyzed by bisulfite DNA sequencing. PCR products were cloned into TOPO TA Cloning vector (Invitrogen) and five clones from each sample were sequenced. Open circle: Unmethylated CpG site; Closed circle: methylated CpG dinucleotide.
(PDF)

Figure S3 **Re-silencing of** ***DLC1*** **and** ***E-Cadherin*** **in HeLa cell after 5-Aza-dC treatment.** HeLa cell was treated with 5 uM 5-Aza-dC for 96 hrs (Day 4) and allowed to recover in drug free culture medium (Day 8 and Day 12). Expression of hypermethylated genes, *DLC1* and *E-Cadherin* were monitored by semi-quantitative RT-PCR. Unmethylated *GSTP1* gene and a house keeping gene, *GAPDH* were served as controls.
(PDF)

Figure S4 **Enrichment of H3K4-me3 in** ***DLC1*** **promoter upon 5-Aza-dC treatment.** SMMC-7721 was treated with 10 uM 5-Aza-dC for 96 hrs. ChIP assay was performed with specific antibody against H3K4-tri-methylation (Upstate). Relative enrichment of H3K4-me3 in 5-Aza-dC treated cells was determined by Q-PCR.
(PDF)

Table S1 **PCR primers and TaqMan Probes.**
(PDF)
