# Supplementary material for: Transcriptional Repressive H3K9 and H3K27 Methylations Contribute to DNMT1-Mediated DNA Methylation Recovery
Source: PLoS One. 2011 Feb 8;6(2):e16702. doi: 10.1371/journal.pone.0016702 (PMC3035659; doi:10.1371/journal.pone.0016702)
Supplement: Figure S1 — Position of primers used in expression and epigenetic studies. Positions indicated above are reference to the putative DLC1 transcription start site (TSS) according to the Genebank database NM_006094. (PDF) [file pone.0016702.s001.pdf]

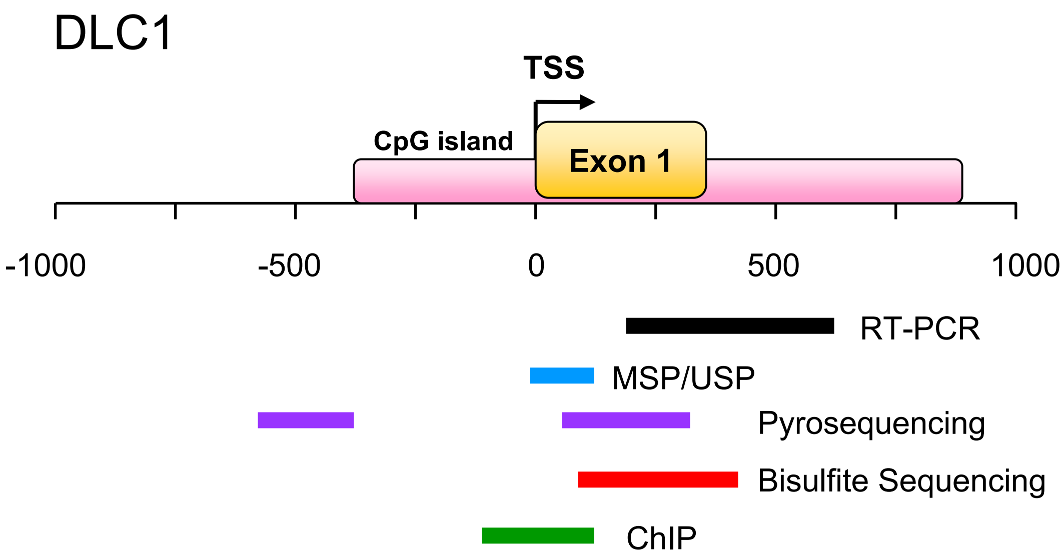

CpG Island: -387 to +765
Exon 1: +1 to +332
RT-PCR: +192 to +649
MSP: -31 to +147
USP: -28 to +144
Pyrosequencing: -579 to -386; +20 to +311
Bisulfite sequencing: +45 to +336
ChIP: -113 to +117
