# Supplementary figures and images for: Transcriptional Repressive H3K9 and H3K27 Methylations Contribute to DNMT1-Mediated DNA Methylation Recovery
Source: PLoS One. 2011 Feb 8;6(2):e16702. doi: 10.1371/journal.pone.0016702 (PMC3035659; doi:10.1371/journal.pone.0016702)

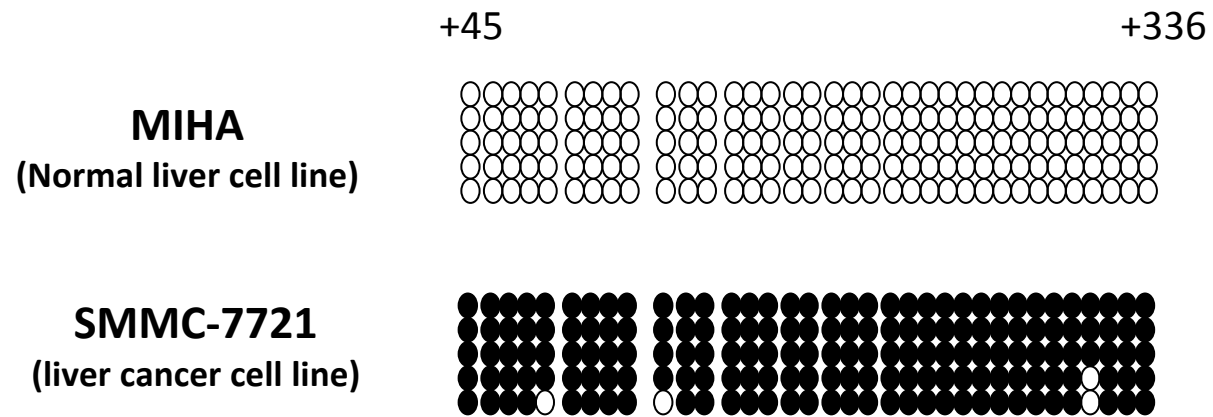

- methylated CpG
- Unmethylated CpG

Supplement: Figure S2 — DLC1 methylation in normal liver cell line (MIHA) and liver cancer cell line (SMMC-7721). DNA methylation at +45 to +336 position of DLC1 gene was analyzed by bisulfite DNA sequencing. PCR products were cloned into TOPO TA Cloning vector (Invitrogen) and five clones from each sample were sequenced. Open circle: Unmethylated CpG site; Closed circle: methylated CpG dinucleotide. (PDF) [file pone.0016702.s002.pdf]

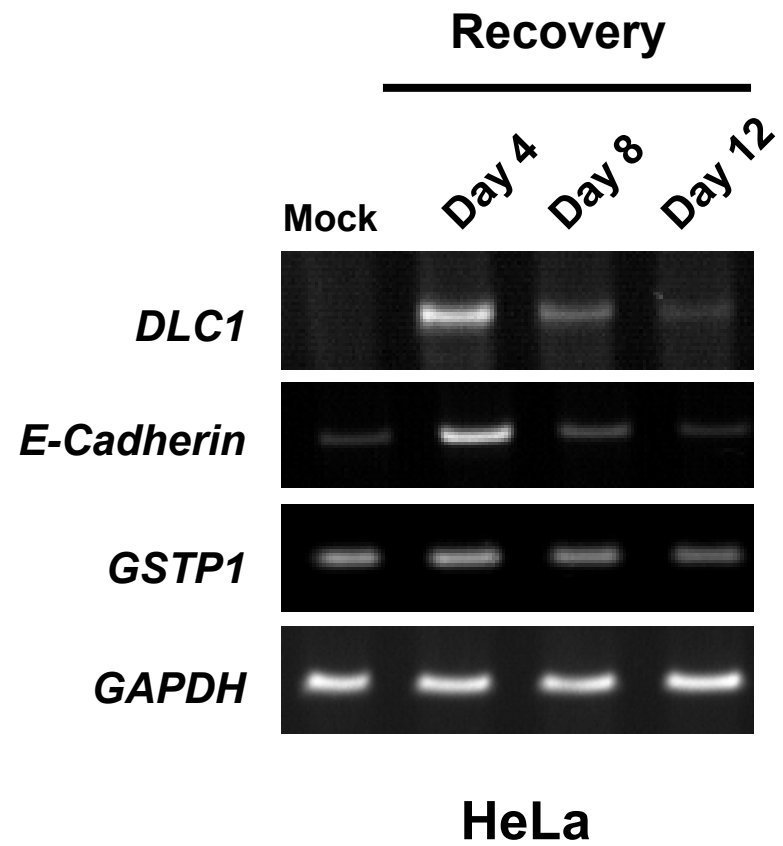

Supplement: Figure S3 — Re-silencing of DLC1 and E-Cadherin in HeLa cell after 5-Aza-dC treatment. HeLa cell was treated with 5 uM 5-Aza-dC for 96 hrs (Day 4) and allowed to recover in drug free culture medium (Day 8 and Day 12). Expression of hypermethylated genes, DLC1 and E-Cadherin were monitored by semi-quantitative RT-PCR. Unmethylated GSTP1 gene and a house keeping gene, GAPDH were served as controls. (PDF) [file pone.0016702.s003.pdf]

# SMMC-7721

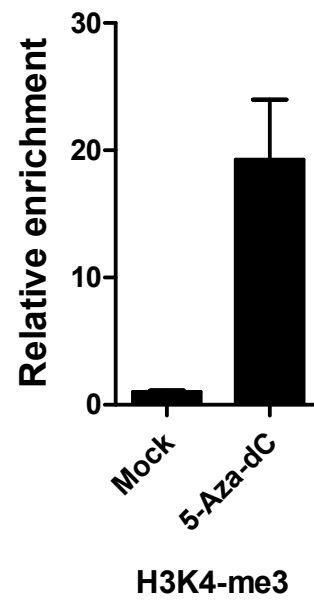

Supplement: Figure S4 — Enrichment of H3K4-me3 in DLC1 promoter upon 5-Aza-dC treatment. SMMC-7721 was treated with 10 uM 5-Aza-dC for 96 hrs. ChIP assay was performed with specific antibody against H3K4-tri-methylation (Upstate). Relative enrichment of H3K4-me3 in 5-Aza-dC treated cells was determined by Q-PCR. (PDF) [file pone.0016702.s004.pdf]
