# Supplementary material for: Transcriptional Repressive H3K9 and H3K27 Methylations Contribute to DNMT1-Mediated DNA Methylation Recovery
Source: PLoS One. 2011 Feb 8;6(2):e16702. doi: 10.1371/journal.pone.0016702 (PMC3035659; doi:10.1371/journal.pone.0016702)
Supplement: Table S1 — PCR primers and TaqMan Probes. (PDF) [file pone.0016702.s005.pdf]

| RT-PCR | Forward primer | Reverse priemr |
|---|---|---|
| DLC1 | AGCAAGGATGCGTTGAGG | CACCTCTTGCTGTCCCTTTG |
| E-Cadherin | CGACCCAACCCAAGAATCTA | GCTGGCTCAAGTCAAAGTCC |
| GSTP1 | ACCTCCGCTGCAAATACATC | GGCTAGGACCTCATGGATCA |
| p16INK4a | CAGGTGGGTAGAAGGTCTGC | GGCTCCTCATTCCTCTTCCT |
| uPA | CTGCTATGAGGGGAATGGTC | GGTTCTCGATGGTGGTGAAT |
| KRT19 | TTTGTGTCCTCGTCCTCCTC | AGAGCCTGTTCCGTCTCAAA |
| GAPDH | ACGCATTTGGTCGTATTGGG | TGATTTTGGAGGGATCTCGC |

| Real-time PCR | TaqMan Gene Expression Assays ID |
|---|---|
| DLC1 | Hs00931487_m1 |
| HPRT | Hs99999909_m1 |

| MSP | Forward primer | Reverse priemr |
|---|---|---|
| DLC1 MSP | TTTAAAGATCGAAACGAGGGAGCG | CCCAACGAAAAAACCCGACTAACG |
| DLC1 USP | TTTTTTAAAGATTGAAATGAGGGAGTG | AAACCCAACAAAAAAACCCAACTAACA |

| Bisulfite DNA sequencing | Forward primer | Reverse priemr |
|---|---|---|
| DLC1 | GTTTTTAGTTAGGATATGGT | ACTTCT TTCTACACATCAAACAC |

| Pyrosequence | Forward primer | Reverse priemr | Extension primer |
|---|---|---|---|
| DLC1 (5'-UTR) | AGTGAAAGTTTAATTTTTTTAAGGTAG | 5'-Biotin-CAAAAATCACAAAACATTTACTT | GTTTTTATAGATTTGGAAATATTGATT |
| DLC1 (1st Exon) | GTTTTTAGTTAGGATATGGT | 5'-Biotin-ACTTCTTTCTACACATCAAACAC | GTTTTTAGTTAGGATATGGT |

| ChIP | Forward primer | Reverse priemr |
|---|---|---|
| DLC1 | AGAACAGCCCGTCGCTAAG | AGGGAGTTGGGCGAGAAGT |
